# Pylorus-Preserving Pancreatoduodenectomy. Experience in 20 Patients

**DOI:** 10.1155/1991/52435

**Published:** 1991

**Authors:** J. Lerut, P. J. Luder, L. Krähenbiühl, P. H. Gertsch, L. H. Blumgart

**Affiliations:** Department of Visceral and Transplantation Surgery Inselspital University Hospital Berne 3010 Switzerland

## Abstract

Twenty patients underwent a pylorus-preserving pancreatoduodenectomy for benign or malignant periampullary and pancreatic disease. Eighteen patients had a partial and two patients a total pancreatectomy. There were 19 elective and 1 emergency operations.

Post-operative mortality was 4% (1/20 patients) and the median follow up was 31 months (range, 15– 75 months), during which period 8 patients with a malignant disease died.

Pylorus-preserving pancreatoduodenectomy did not compromise survival in ampullary cancer.

One patient developed a marginal ulcer during the study period and one of twelve patients, examined by technetium scintigraphy (done more than 3 months after the procedure), had delayed gastric emptying. Two patients presented with a gastric retention as the first sign of recurrent pancreatic cancer.

The result of the operation was judged as excellent in 7 patients, good in 8 and as bad in only 2 of the 17 patients who survived more than *6 months* . Body weight was studied in 15 patients surviving more than *one year* after operation; five patients had gained weight, two had lost weight and in 8 there was no difference.

Pylorus-preserving pancreatoduodenectomy seems to be a valuable alternative in the treatment of patients with benign and selected malignant pancreaticobiliary disease.


*HPB Surgery*, 1991, Vol. 4, pp. 109–119
Reprints available directly from the publisher
Photocopying permitted by license only

© 1991 Harwood Academic Publishers GmbH
Printed in the United Kingdom

### PYLORUS-PRESERVING PANCREATODUODENECTOMY. EXPERIENCE IN 20 PATIENTS

J. LERUT, P.J. LUDER, L. KRÄHENBÜHL, P.H. GERTSCH and L.H. BLUMGART

*Department of Visceral and Transplantation Surgery, Inselspital, University Hospital, 3010 Berne, Switzerland*

*(Received 13 December 1990)*

Twenty patients underwent a pylorus-preserving pancreatoduodenectomy for benign or malignant periampullary and pancreatic disease. Eighteen patients had a partial and two patients a total pancreatectomy. There were 19 elective and 1 emergency operations.

Post-operative mortality was 4% (1/20 patients) and the median follow up was 31 months (range, 15–75 months), during which period 8 patients with a malignant disease died.

Pylorus-preserving pancreatoduodenectomy did not compromise survival in ampullary cancer.

One patient developed a marginal ulcer during the study period and one of twelve patients, examined by technetium scintigraphy (done more than 3 months after the procedure), had delayed gastric emptying. Two patients presented with a gastric retention as the first sign of recurrent pancreatic cancer.

The result of the operation was judged as excellent in 7 patients, good in 8 and as bad in only 2 of the 17 patients who survived more than *6 months*. Body weight was studied in 15 patients surviving more than *one year* after operation; five patients had gained weight, two had lost weight and in 8 there was no difference.

Pylorus-preserving pancreatoduodenectomy seems to be a valuable alternative in the treatment of patients with benign and selected malignant pancreaticobiliary disease.

KEY WORDS: Pancreatic surgery, pancreatic resection, pylorus preservation, gastric emptying, nutrition, periampullary cancer

### INTRODUCTION

Kausch and Whipple performed the first successful pancreatoduodenal resections (PDR) with preservation of the entire stomach and a gastrojejunostromy was performed for restoration of the gastro-intestinal continuity[4]. Because of the ulcerogenic nature of the operation and because of oncological reasons, partial gastric resection was added to the procedure. Although Watson described pylorus-preserving PDR (PP-PDR) in 1942, it was not until 1978 that the metod was popularized by Longmire[25]. The goal of this latter procedure was to decrease morbidity (a consequence of the reduced gastric reservoir) and thus to improve the nutritional status of the patient. Because of the success of the procedure in benign periampullary diseases[9,26], the indications have been widened to include malignant disease[4,11].

Address correspondence to: PD Dr. Jan Lerut, Department of Visceral and Transplantation Surgery, Inselspital, 3010 Berne, Switzerland

This study analyzes morbidity and postoperative function in a series of 20 patients undergoing PP-PDR during the period May 1984 – June 1989.

### METHODS AND MATERIAL

### *Technique of Pylorus-preserving Pancreatoduodenal Resection*

After extensive Kocherisation of the duodenum and mobilisation of the hepatic colonic flexure, the entire duodenum was inspected. Major inflammatory changes as well as tumor involvement of the hepatoduodenal ligament and/or the first part of the duodenum precluded PP-PDR. The first part of the duodenum was in all, but one, patient transsected about 4 cm distally to the pyloric ring, using a GIA-stapler.

The neurovascular supply of stomach, pylorus and duodenum were preserved. The gastroduodenal artery was divided at its junction with the hepatic artery; the right gastric artery, if present, was also divided. This approach allowed adequate dissection of the hepatoduodenal ligament in malignant diseases. The gastroepiploic artery was divided at its origin from the pancreaticoduodenal artery and the corresponding vein was divided at its entrance into the gastroepiploic trunk. The gastroepiploic vessels were thus preserved along the greater gastric curvature. After freeing the stomach, the vascularisation of the duodenal stump may sometimes be compromised; although a bluish appearance of the duodenum does not automatically preclude the pylorus-preserving procedure. In one patient a more proximal resection of the duodenal stump had to be done clearly for this reason. Histological examination showed no evidence of tumor in the duodenal transsection margin in any of the patients with malignant disease.

Restoration of alimentary tract continuity was done using two different methods. In fifteen patients a single jejunal loop was used for anastomosis to the pancreatic remnant, the hepatic duct and the duodenum. Pancreatojejunostomy was covered nine times by the greater omentum in order to separate this site from the biliary and gastric anastomoses. In 5 patients gastrointestinal reconstruction was performed using a split loop technique[8], where the duodenum was anastomosed in an end-to-end fashion to the jejunum. The pancreatic remnant and bile duct were anastomosed to a second Roux-Y jejunal limb. Because of poor quality of the pancreatic remnant an invagination technique was used twice.

The duodenojejuenostomy was performed using a single layer of interrupted or running sutures Maxon/PDS 4/0. The jejunal loop(s) were fixed to the irrespective openings in the transverse mesocolon in order to avoid internal herniation and gastric outlet obstruction. A parapancreatic penrose drainage was inserted in all partially resected patients.

Twelve men and eight women (median age 59 years; range 27–71 years) underwent PP-PDR for pancreatic adenocarcinoma[8], pancreatic acinar carcinoma[1], ampullary cancer[3], cholangiocarcinoma[2], benign insulinoma[2], pancreatic cystadenoma[2] and chronic pancreatitis[1]. One emergency procedure was carried out because of bleeding from a duodenal leiomyoma.

Eighteen patients underwent partial pancreatectomy. One patient with a pancreatic adenocarcinoma and another with a pancreatic cystadenoma were treated by total pancreatectomy.

All patients were reviewed at intervals of 3–6 months for at least 15 months or until death. The median follow up was 31 months (range, 15–75 months). The postoperative results were judged using a detailed questionnaire designed to assess nutritional habits, postgastrectomy syndromes and symptoms, bowel movements, meal capacity, weight change, subjective appreciation and quality of life based on Visick's grading.

Gastric emptying was evaluated after full recovery from the operation (3 to 6 months postoperatively) by solid and liquid meal scintigraphy in 12 patients.

Ulcer formation was assessed by interrogatory into respect to peptic ulceration in all survivors, by endoscopy in eight patients, by upper-GI-series in four patients and at autopsy in one patient.

### RESULTS

None of 18 patients treated by partial PDR died; one of the two patients undergoing total pancreatectomy for pancreatic cystadenoma died of a pulmonary embolism on the first postoperative day.

The hospital mortality for the whole group was 4% (1/20 pat.). Eight patients (40%) experienced a postoperative complication: temporary pancreatic fistula$^{5}$, pneumonia$^{1}$, wound infection$^{1}$ and fatal pulmonary embolism$^{1}$.

The median duration of nasogastric drainage for all patients was 9.2 days (range, 2 to 22 days). Patients without pancreatic fistula had a median gastric drainage of 5.6 days (range, 2 to 7 days); those who developed a pancreatic fistula required a longer gastric drainage (median of 8.5 days; range, 13 to 22 days).

The mean survival of the nine patients who underwent a resection for a pancreatic cancer is 13.5 months. Six patients died after 5,10,10,13,17 and 30 months respectively. The two latter patients had no lymph node involvement at surgery. Three "lymph node free" patients remain alive at 15, 15 and 29 months.

One of two patients undergoing resection for cancer of the distal third of the bile duct died of a recurrence at 9 months, the other patient is alive at 17 months despite lymph node involvement at resection.

The three patients undergoing resection for ampullary carcinoma are surviving disease free 23, 42, and 45 months after surgery; all 3 patients were free of lymph node metastases at surgery.

The five patients surviving after resection for benign disease are alive at 17, 51, 69, 73 and 74 months.

The upper GI-tract was controlled by endoscopy in eight patients, by upper GI-series in four patients and at autopsy (one year after PDR) in one patient. All, but one, patient had normal investigations. This female patient had to be rehospitalized two years postoperatively because of a bleeding stomal ulcer; this ulcer healed after medical treatment with omeprazole. Review of her operative record revealed that the duodenum had been transsected at a distance less than 1.5 cm from the pyloric ring.

Technetium-gastric emptying studies for solid and fluid meals were carried out in 12 patients. Emptying of the stomach was normal in 9, delayed in 2 and accelerated in one patient. One patient with a delayed and one with an accelerated emptying had had a temporary pancreatic fistula after the procedure. Two patients, presenting with postprandial gastric fulness and one patient, with the sensation of rapid gastric emptying, had normal scintigraphic gastric transit times.

One patient developed a disturbance of gastric emptying 20 months postoperatively due to local recurrence of pancreatic cancer. Two patients however, were rehospitalized 10 and 12 months after resection for pancreatic head cancer because of a gastric retention in the absence of endoscopic and radiological gastric outlet obstruction. The *gastric emptying disturbance* was the *first sign of recurrent pancreatic cancer* in both these patients.

Seventeen patients surviving more than *6 months*, were assessed on a Visick score. The result was judged excellent (grade I) in 7, good (grade II) in 8 and bad (grade III) in 2 patients.

Weight changes were studied in fifteen patients surviving more than *one year*. In comparison to preoperative weight, five patients gained weight (3, 5, 5, 8 and 12 kg), eight remained unchanged. One patient, with early recurrence of pancreatic cancer had a significant weight loss. Another patient who underwent a PP-PDR for invalidating alcoholic chronic pancreatitis initially presented with a weight gain of five kg, but his eventual weight was seven kg less than his preoperative weight.

### DISCUSSION

The original one-stage Whipple procedure included preservation of the stomach[4]. Fifty to seventy percent gastric resection (with or without truncal vagotomy) was later added in order to fulfil the principles of oncological resection of malignant disease and to reduce the incidence of ulcer formation. However the creation of this reduced gastric reservoir was responsible for significant late morbidity[4,7,17].

In 1942 Watson introduced PDR with preservation of the innervated stomach, the pyloric ring and the first part of the duodenum[4]. The goal of this procedure was to improve nutritional status and to reduce or eliminate the clinical sequelae of the reduced gastric reservoir. Although the concept was sound, it took more than 30 years before this operation became popularized[25]. The so called "Watson-Longmire" procedure was initially reintroduced in clinical practice for the treatment of benign pancreatic diseases and for certain duodenal tumors. As experience in its use increased it was possible to extend the indications in selected cases of malignant periampullary diseases[3,11,20,29]. The value of PP-PDR in pancreatic resectional surgery can only be assessed after the following questions have been addressed: (a) are postoperative morbidity and mortality decreased, (b) is the rate of stomal ulcer formation reduced, (c) are gastrointestinal function and the nutritional status improved and (d) is the operation justified in cancer patients.

The *operative mortality* of PDR, has been reduced during the last decade beneath 10% and in many specialised centers even below 5% (4, 12, 16, 27, 29). Similar figures have been reported for PP-PDR (3, 12, 17, 20, 26). The latter operation is somewhat simpler to perform since the gastroenterostomy is replaced by a duodenojejunostomy. Leakage of this anastomosis is extremely rare although marginal vascularization of the duodenal stump during surgery has been described[3,14].

In view of experience with the classical Whipple operation, a fear existed that PP-PDR would increase the risk of *stomal ulcer* formation[4]. The ulcer rate following PDR ranges from 6 to 20% with a mean time of ulcer development at 15 months. Eighty percent of these ulcers develop during the first three postoperative months, but they may appear up to 3 years after pancreatic resection[4,17]. A literature survey of well documented PP-PDR series reveals an ulcer incidence of 8.4% (3,9,10,12,13,14,15,17,18,20,26) (Table 1). Not all reported series have however a minimal follow up of 3 years[12,13,19]. If one excludes the series of Gebhardt[10], reporting a very high incidence (62%) of ulcer formation after PP-PDR (with pancreatic duct occlusion) carried out in patients with chronic alcoholic pancreatitis, this incidence drops to 5,4%. The incidence of ulcer formation after PP-PDR thus seems currently no higher than after PDR. The ulcer rate after pancreatic resection must moreover be interpreted with caution since pancreatic insufficiency due to inadequate pancreatic enzyme replacement stimulates gastric acid secretion and favours ulcer formation[12,26]. The occurrence of ulcer in patients with chronic pancreatitis[10,13,18], in patients with total pancreatectomy[13,17] or in patients with PDR and pancreatic duct occlusion[10] and in patients undergoing adjuvant postoperative radiotherapy[3,17,20] must also be interpreted in this context.

If ulcers occur, the majority of them respond to adequate medical treatment; vagotomy with pyloroplasty or even partial gastric resection are only rarely necessary[3,12,13,17,18].

**Table 1** Ulcer formation after PP-PDR (in well documented series).

| *Author* | *Ref.* | *N* | *Patients with chronic pancreatitis* | *Remarks ulcer patients* | *Therapy* | |
|---|---|---|---|---|---|---|
| GEBHARDT | (10) | 12/18* | 18 | all alcoholics | | |
| FLAUTNER | (9) | 0/37°* | 37 | | | |
| BRAASCH | (3) | 5/85 | 28 | 1 RTH | medical | 3 |
| | | | | | transthoracic truncal vagotomy | 1 |
| | | | | | relaparotomy | 1 |
| HUNT | (13) | 3/15 | 5 | 1 Tot. Pa. | medical | 3 |
| GRACE | (12) | 1/34 | ? | | gastric resection | 1 |
| MOSCA | (14) | 0/30 | 8 | | | |
| ITANI | (14) | 0/7 | 1 | | | |
| KIM | (15) | 0/13+ | 6 | | | |
| PATTI | (21) | 0/10 | 2 | | | |
| PARTENSKY | (20) | 1/36 | 6 | 1 RTH | medical | 1 |
| MC AFEE | (17) | 4/31 | 12 | 1 Tot. Pa. | vagotomy pyloroplasty | 1 |
| | | | | | medical | 3 |
| MOREL | (18) | 4/33 | 20 | | reoperation | 2 |
| | | | | | gastric resection | 1 |
| | | | | | medical | 1 |
| OWN SERIES | | 1/19 | 1 | | medical | 1 |
| Total | | 31/368 (8.4%) [19/350 (5.4% if GEBHARDT'S series excluded)] | | | | |

N = ulcer formation in relation to postoperative survivors
° = pancreatic duct occlusion
* = pancreato-gastrostomy
+ = Duodenum I–IV reanastomosis
Tot Pa: total pancreatectomy
RTH: postoperative adjuvant radiotherapy

The key factors in the low ulcer incidence after PP-PDR relate to endocrine and exocrine gastroduodenal function[14,15,21,24]. The feedback mechanism between parietal and gastrin producing cells being maintained after PP-PDR, although during the early postoperative period there may be a temporary increase of basal and maximal acid output with a decrease of gastrin secretion. These changes are probably due to initial inadequate recovery of the stomach; after about a year, gastric acid output and gastrin production again fall to preoperative levels[15]. No difference in postprandial gastrin and secretin plasma levels after PP-PDR as compared to healthy controls can be found[24]. Normal secretion levels ensure appropriate pancreatic secretion which in turn helps normal digestion. Normal gastrin level together with adequate acid secretion and normal sized stomach allow intake and digestion of normal amounts of food.

The preserved pyloric ring reduces or prevents the, physiological, enterogastric reflux which is a factor in preventing ulcer formation. An even greater role in ulcer prevention is played by the duodenal bulb, which releases gastric emptying and acid secretion regulating hormones; Brunner's glands may also play a role in neutralisation of gastric acid[15]. The fact that the only patient in these series presenting with a stomal ulcer, had a very short part of duodenal bulb preserved (to achieve the duodenojejunostomy) may be in favour of this hypothesis. A maximal length of duodenum should thus be preserved during PP-PDR. This objective is facilitated by the use of a stapling device during duodenal transsection.

The further the distance from the pylorus, the less is the buffering capacity of the jejunum. It is thus preferable to anastomose the duodenum to as proximal a loop of jejunum as possible; this being especially important if a split loop technique is used for gastrointestinal reconstruction[8,18].

A delay in *gastric emptying*, defined as the necessity for gastric drainage for more than 7 days, occurs in up to 50% of patients after PP-PDR[4,14,28]. The delayed gastric emptying has a multifactorial etiology and may be due to devascularization of the duodenal bulb, to operative trauma affecting the vagal innervation of the antropyloric pump or to modification of the complex endocrinological interactions between stomach and intestine. However this complication is usually temporary and the mainstay of therapy is gastric decompression with transpyloric enteral feeding[4,28]. Stimulation of gastric motility is not useful. Exceptionally, pyloroplasty, gastric bypass or even partial gastric resection are necessary[13,14,28]. Prophylactic gastrostomy may be useful, occasionally if postoperative problems such as pancreatic fistula are anticipated. Prolonged gastric retention is indeed frequently the expression of an intraabdominal complication. It is important to recognize that about 60% of pancreatic cancer patients have delayed preoperative gastric emptying, a phenomenon which may be due to perineural infiltration[28].

Late delayed gastric emptying, in the absence of an organic lesion, may be the first indicator of recurrent malignancy during the postoperative period[21], *vide supra*.

Detailed gastric emptying studies, using scintigraphy and upper GI-series, have essentially shown normal gastric emptying and pyloric functioning after PP-PDR[4,6,13,21,26] (Figure 1). Emptying of solids show, as after partial gastrectomy, great variability with liquids in particular emptying more rapidly[6,24]. The intestinal transit is normal or little accelerated after PP-PDR[21]. The results of these functional examinations may be related to the fact that the pacemaker, located within the first

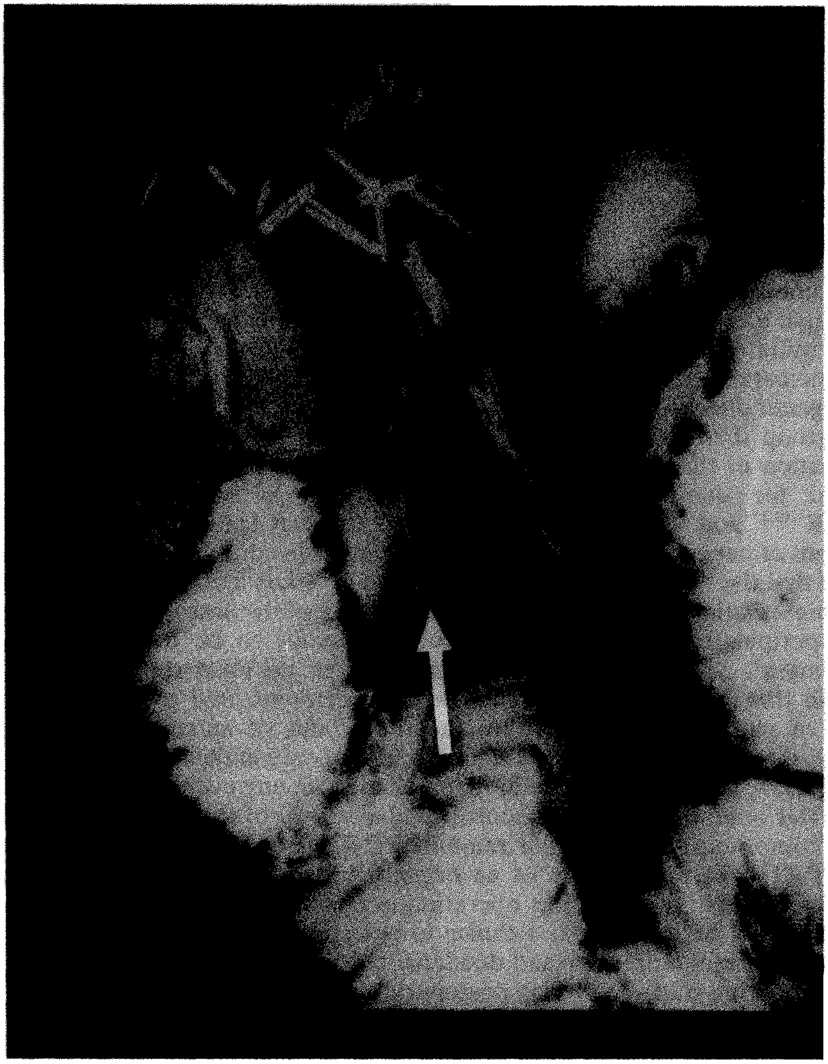

**Figure 1** Upper GI-series showing normal gastric emptying and functioning of pyloric ring (↗) after PP-PDR.

centimeter of the duodenum (regulating gastric emptying and playing a major role in the regulation of small bowel motility) is spared.

The preservation of the antropyloric segment is important in sieving and grinding of solid foods[21]. The gut hormone producing cells are more densily distributed in the duodenum and the proximal jejunum. Direct stimulation of this area by gastric juice and food will lead to a better coordination of pancreatic, biliary and gastric secretions leading to better mixing of food and digestive juices and to alkalinisation of the postpyloric intestine[24]. Less marginal ulceration and better absorption of nutrients may thus be expected after PP-PDR. The beneficial effect of the duodenum on hormonal and nutritional status of partially pancreatectomized patients is well demonstrated in Beger's procedure in which the head of the pancreas is resected preserving the complete duodenal arch[1]. Sparing of the first and fourth parts of the duodenum during pancreatectomy with subsequent reanastomosis could therefore be a further step in the amelioration of the nutritional status of the pancreatectomized patient[23].

Lastly, elimination of vagotomy, with its inherent risk of diarrhoea and enhanced pancreatic insufficiency, is together with the better maintenance of gastrointestinal physiology, another important factor in the improvement of the *nutritional status* of the patient undergoing PP-PDR. About 80% of patients have normal meal capacity and weight gain is observed in the majority of the patients[3,12,14,26].

The initial success of the PP-PDR in the resectional treatment of benign biliopancreatic diseases led to a more frequent use of the method in the treatment of *malignant duodenal and periampullary diseases*. PP-PDR is only justified in the latter group if tumor free transsection margins are obtained. A frozen section examination of the duodenal stump should be made to exclude tumor invasion. Survival for duodenal and ampullary cancers is not compromised after PP-PDR[4,19,20] and it has also been reported to be a valuable alternative in the treatment of pancreatic head cancer[11]. Justification for this view relies on accepting the fact that the "only difference" between the PP-PDR and the standard PDR lies in the preservation of the tissue of the lesser curve of the antropyloric segment and of the peripyloric region which are known, usually to be free of lymph node involvement[5]. Some observations have however demonstrated positive lymph nodes in this region and recent reports have demonstrated proximal intramural spread of pancreatic cancer within the duodenum or antrum of the stomach. PP-PDR may therefore compromise the chance of cure in these resectable pancreatic cancers[2,22]. Large pancreaticoduodenal tumors, in any event, are a contraindication to this operation since the neurovascular supply of the antropyloric segment will be compromised after tumor removal[11,22]. The occurrence of gastric retention after PP-PDR in the absence of an organic gastroduodenal lesion, is a further argument against the use of this operation in pancreatic cancer.

PP-PDR has become the first choice in resectional treatment of benign and of selected malignant ampullary and duodenal cancers. The observation that delayed gastric emptying during the late postoperative period may be the first sign of a recurrent pancreatic cancer in the absence of an organic gastroduodenal lesion as well as the observation that some pancreatic tumors may have a more proximal intramural carcinoma spread should alert us about the performance of PP-PDR for pancreatic cancer and will often mandate that a classical procedure should be carried out.

### *References*

1. Beger, H.G. and Buchler, M. (1990) Duodenum-preserving resection of the head of the pancreas in chronic pancreatitis with inflammatory mass in the head. *World J. Surg.*, **14**, 83–87
2. Boerma, E.J. and Coosemans, J.A.R. (1990) Non-preservation of the pylorus in the resection of pancreatic cancer. *Br. J. Surg.*, **77**, 299–300
3. Braasch, J.W., Deziel, D., Rossi, R. *et al.* (1986) Pyloric and gastric preserving pancreatic resection. Experience with 87 patients. *Ann. Surg.*, **10**, 411–418

4. Braasch, J.W. (1988) Pancreatoduodenal resection. *Curr. Probl. Surg.*, Year Book Med. Publ., **25**, 5
5. Cubilla, A.L. and Fitzgerald, P.J. (1980) Surgical pathology aspects of cancer of the ampulla-head-of-pancreas region. In: Fitzgerald, P.J., Morrison A.B. (eds). *The Pancreas.* International Academy of Pathology. Williams and Wilkins, 67–82
6. Fink, A.S., De Souza, L.R., Mayer, E.A. *et al.* (1988) Long-term evaluation of pylorus preserving during pancreaticoduodenectomy. *World J. Surg.*, **12**, 663–670
7. Fish, J.C., Smith, L.B. and Williams, R.D. (1969) Digestive function after radical pancreaticoduodenectomy. *Am. J. Surg.*, **117**, 40–45
8. Funovic, S., Zöch, G., Wenzl, E. and Schulz, F. (1987) Progress in reconstruction after resection of the head of the pancreas. *S.G.O.*, **164**, 545–548
9. Flautner, L., Tihanyi, T. and Szecseny, A. (1985) Pancreatogastrostomy: an ideal complement to pancreatic head resection with preservation of the pylorus in the treatment of chronic pancreatitis. *Am. J. Surg.*, **150**, 608–611
10. Gebhardt, V.C., Gall, F.P., Rosch, W. and Schackert, H.K. (1982) Anastomosenulkus nach Whipplescher Operation mit Magenerhaltung. *Zentralbl. Chir.*, **107**, 952–958
11. Grace, P.A., Pitt, H.A. and Longmire, W.P. (1986) Pancreatoduodenectomy with pylorus preservation for adenocarcinoma of the head of the pancreas. *Br. J. Surg.*, **73**, 647–650
12. Grace, P.A., Pitt, H.A. and Tompkins, R.K., *et al.* (1986) Decreased morbidity and mortality after pancreaticoduodenectomy. *Am. J. Surg.*, **151**, 141–149
13. Hunt, D.R. and McLean, R. (!989) Pylorus-preserving pancreatectomy: functional results. *Br. J. Surg.*, **76**, 173–176
14. Itani, K.M.F., Coleman, R.E., Meyers, W.C. and Akwari, O.E. (1986) Pylorus-preserving pancreatoduodenectomy. A clinical and physiologic appraisal. *Ann. Surg.*, **12**, 655–664
15. Kim, H.C., Suzuki, T., Kajiwara, T. *et al.* (1987) Exocrine and endocrine stomach after gastrobulbar preserving pancreatoduodenectomy. *Ann. Surg.*, **12**, 717–727
16. Lerut, J., Gianello, P., Otte, J.B. and Kestens, P.J. (1984)Pancreaticoduodenal resection. *Ann. Surg.*, **199**, 432–437
17. Mc Afee, M.K., Van Heerden, J.A., Adson, M.O. *et al.* Is proximal pancreatoduodenectomy with pyloric preservation superior to total pancreatectomy. *Surgery,* **105**, 347–351
18. Morel, P.H., Mathey, P., Corboud, H. *et al.* (1990) Pylorus-preserving duodenopancreatectomy: Long-term complications and comparison with the Whipple procedure. *Wild J. Surg.*, **12**, 642--647
19. Newmann, K.D., Braasch, J.W., Rossi, R.L. and O'Campo-Gonzalez, S. (1983) Pyloric and gastric preservation with pancreatoduodenectomy *Am. J. Surg.*, **145**, 152–156
20. Partensky, C., Champetier, P. and Faure, J.L. (1988) Peut-on élargir les indications de la conservation pylorique au cours de la duodénopancréatectomie? *Ann. Chir.*, **42**, 313–317
21. Patti, M.G., Pellegrini, A. and Way, L.W. (1987) Gastric emptying and small bowel transit of solid food after pylorus-preserving pancreaticoduodenectomy. *Arch. Surg.*, **122**, 528–531
22. Sharp, K.W., Ross, C.H.B., Halter, B.A. *et al.* Pancreatoduodenectomy with pyloric preservation for carcinoma of the pancreas: a cautionary note. *Surgery,* **105**, 645–653
23. Suzuki, T., Imamura, M., Kajiwara, T. *et al.* (1988) A new method of reconstruction after pylorus-preserving pancreatoduodenectomy. *World J. Surg.*, **12**, 645–650
24. Takada, T., Yasuda, H., Shikata, J. *et al.* Postprandial plasma gastrin and secretin concentrations after a pancreatoduodenectomy. *Ann. Surg.*, **210**, 47–51
25. Traverso, L.W. and Longmire, W.P. (1978) Preservation of the pylorus in pancreaticoduodenectomy. *S.G.O.*, **146**, 959–962
26. Traverso, L.W. and Longmire, W.P. (1980) Preservation of the pylorus in pancreaticoduodenectomy. A follow up evaluation. *Ann. Surg.*, **192**, 306–310
27. Trede, M., Schwall, G. and Saeger, H.D. (1990) Survival after pancreatoduodenectomy: 118 consecutive resections without an operature mortality. *Ann. Surg.*, **211**, 447–458
28. Warshaw, A.L. and Torchiouna, D.L. (1985) Delayed gastric emptying after pylorus-preserving. *S.G.O.*, **160**, 1–4
29. Warshaw, A.L. and Swanson, R.S. (1988) Pancreatic cancer in 1988. *Ann. Surg.*, **208**, 541–553

### INVITED COMMENTARY

Our experience with approximately 170 pyloric-preserving pancreatoduodenectomies for benign (chronic pancreatitis) and malignant disease (ampullary, distal bile duct and pancreatic tumors) is in agreement with the findings of this report. The operation is simplified by avoiding a gastrectomy, gastric emptying and gastric capacity are maintained, marginal ulceration is uncommon and post-gastrectomy syndromes are extremely rare. It is our impression that in patients that have received radiation therapy following resection the incidence of marginal ulceration may be increased.

We are currently using the pyloric-preserving pancreatoduodenectomy for the management of most patients with pancreatic carcinoma unless ischemia of the antropyloric and duodenal area is noted at the time of surgery. It is our bias that in patients with pancreatic carcinoma, the additional tissue removed by a gastrectomy (antrum, pylorus, nodes of the lesser and greater curvature), if involved by tumor, would not improve the survival by a standard pancreatoduodenectomy as we would probably be dealing with incurable disease. Our 5 year adjusted survival in patients with ampullary, biliary and pancreatic carcinoma treated by PPPD is 43%, 53%, and 18% respectively. Microscopic involvement of margins were an important prognosis factor. However, the margin most commonly involved in pancreatic cancer was the posterior aortic margin rather than the duodenal line of transection or the line of transection in the neck of the pancreas. I praise the authors of this paper for having studied their patients very well and for having increased the information available in regard to the value and limitations of this procedure.

Ricardo L. Rossi
Lahey Clinic Medical Center

### INVITED COMMENTARY

This review of pyloric preserving pancreatoduodenectomy provides a careful documentation of the clinical setting in which this procedure is presently used as well as the expected short term results with this procedure. Whether or not pylorus preserving Whipple procedures represent a significant advance over the more conventional type is yet to be determined but the comparable short term results warrant continued usage.

Theoretical or practical advantages of the procedures are threefold: 1) the procedure is often technically simpler and faster than the conventional type, 2) it seems teleologically better to preserve as much gastrointestinal digestive or reservoir function as possible, and, 3) the procedure may have some real long term advantages such as less ulcer disease or better nutrition. Disadvantages of the procedure are that 1) it is one less time tested than the more conventional resection, 2) what seem to be anatomical advantages may not be physiological advantages and, 3) the procedure could limit the surgical margin for neoplastic disease. None of the potential advantages or disadvantages have been proved, nor have there been retrospective analyses that have provided much insight into any of these questions. It seems that pyloric preserving procedures may be associated with worse early gastric emptying and better long term emptying, but this has never been adequately studied. Most of these questions, however, do seem answerable, and it is doubtful that any one center could provide those answers because of the relatively small number of Whipple prrocedures performed at any one center and the mixture of diseases. Therefore, this subject seems a tremendously fruitful one for cooperative, prospective studies!

William C. Meyers
Chief of Gastrointestinal Surgery
Duke University Medical Center
Durham, North Carolina 27710 USA

